# Flavonoid Metabolic Profiles and Gene Mapping of Rice (*Oryza sativa* L.) Purple Gradient Grain Hulls

**DOI:** 10.1186/s12284-022-00589-x

**Published:** 2022-08-08

**Authors:** Fantao Zhang, Limin Yang, Wenxue Huang, Xiangdong Luo, Jiankun Xie, Biaolin Hu, Yaling Chen

**Affiliations:** 1 Laboratory of Plant Genetic Improvement and Biotechnology, College of Life Sciences, Jiangxi Normal University, No 99, Ziyang Road, Nanchang, 330022 Jiangxi China; 2 Rice Research Institute, Jiangxi Academy of Agricultural Sciences/National Engineering Laboratory for Rice (Nanchang), No 1738, Liangtangbei Road, Nanchang, 330200 Jiangxi China

**Keywords:** Rice, Purple gradient grain hull, Cyanidin, BSA-seq, Flavonoids

## Abstract

Rice (*Oryza sativa* L.) grain hull color is an easily observable trait and regarded as a crucial morphological marker in rice breeding. Here, a purple gradient grain hull mutant (*pg*) was found from natural mutations of a straw-white grain hull rice variety IARI 6184B (*Orzya sativa* L. subsp. *indica*). The color of the mutant grain hulls changed from straw-white to pink, then purple, and finally brownish-yellow. Ultra-performance liquid chromatography-tandem mass spectrometry (UPLC-MS/MS) identified 217 flavonoids, including 18 anthocyanins, among which cyanidin O-syringic acid had the highest concentration in pink (66.2 × 10^6^) and purple (68.0 × 10^6^) grain hulls. The relative contents of hesperetin O-malonyl-hexoside, apigenin derivatives, genistein derivatives, and kaempferol 3-O derivatives were consistently downregulated during *pg* grain hull development. Conversely, 12 anthocyanins were upregulated in colored hulls, and cyanidin 3-O-malonylhexoside was abundant only in pink and purple grain hulls. Moreover, the candidate gene was mapped into a 1.38 Mb region on chromosome 4 through bulked segregant analysis based on deep sequencing (BSA-seq) and gene mapping approaches. These results increased our understanding of anthocyanin biosynthesis in rice grains, helping rice breeders to select new rice varieties with desirable grain traits.

**Supplementary Information:**

The online version contains supplementary material available at 10.1186/s12284-022-00589-x.

## Background

Rice (*Oryza sativa* L.) is one of the most important cereals consumed by nearly half of the world’s population. Rice has various phenotypes and agronomic characteristics, such as seed texture, shape, and pericarp color (Saitoh et al. 2004; Zhu et al. 2011). Some colored varieties with purple leaf sheaths, red pericarp, red leaves, purple stigma, and black hulls have been used in rice breeding as morphological markers for identifying varieties and studying linkages in recent years (Choudhury et al. 2014; Khan et al. 2020). In particular, grain hull color can be used as an extraction material for flavonoids and a marker for identifying male-sterile and restorer lines for mechanized commercial hybrid rice seed production (Tang et al. 2020).

Flavonoids are a large class of biologically active secondary metabolites and are key factors affecting plant color (Lepiniec et al. 2006; Koirala et al. 2016). The biosynthetic pathway of flavonoids has been relatively well elucidated in *Arabidopsis* (Lepiniec et al. 2006). For example, phenylalanine was identified as a flavonoid precursor catalyzed by phenylalanine lyase, cinnamic acid-4-hydroxylase, and 4-coumarate coenzyme A ligase to form *p*-coumarate coenzyme A through a series of reactions (Tohge et al. 2017). Furthermore, the *p*-coumarate coenzyme A (phenylpropanoid primers) and malonyl-CoA (polyketide condensing unit) were further modified by different classes of enzymes into various flavonoid subclasses, including chalcones, flavonols, flavanediols, flavones, proanthocyanidins, and anthocyanins (Tohge et al. 2017; Nabavi et al. 2020). Anthocyanin is an end-product of the flavonoid pathway; however, its accumulation differs among colored rice varieties (Xia et al. 2021). Meanwhile, several flavonoids, upstream of or along other pathways, also dynamically influence anthocyanin metabolism (Saigo et al. 2020). Therefore, it is necessary to determine the dynamic metabolic patterns of pigments in colored rice through the detection, identification, and quantification of flavonoids on a large scale.

Rice pigmentation is regulated by the catabolite activator protein system, a complementary gene system consisting of three different kinds of genes: *C* (chromogen), *A* (activator), and *P* (tissue-specific regulator). Saitoh et al. (2004) first localized the *C* gene, which encodes a transcription factor belonging to the myeloblastosis (MYB) family, on the short arm of chromosome 6 in rice. Then, *OsC1* was cloned using natural rice variants in the same year to produce purple coloration on the leaf sheath, apiculus, and stigma (Nagabhushana and Arjula 2004; Fan et al. 2008; Choudhury et al. 2014). The *Ra1*/*OsB1 OsB2*, *Rb*, and *Rc* genes encode proteins containing the basic helix-loop-helix (bHLH) protein motif that activates downstream genes related to anthocyanin metabolism in rice (Sakamoto et al. 2001; Sweeney et al. 2006). The purple pericarp trait is regulated by *Kala1*, *Kala3*, and *Kala4* genes (Oikawa et al. 2015; Kim et al. 2021). The brown hull repressor inhibitor for *brown furrows 1* encodes the F-box protein OsFBX310, which regulates hull pigment synthesis and deposition (Shao et al. 2012; Xu et al. 2015). Deleting the chalcone isomerase gene *OsCHI* increased hull flavonoid content, showing a golden yellow color (Hong et al. 2012). Several studies have been conducted on the genetics of purple coloration in the leaves, apiculus, and pericarp. However, activators or tissue-specific regulators of purple grain hull traits have not been well identified.

In this study, a purple gradient grain hull mutant (*pg*) was identified from a straw-white grain hull rice variety IARI 6184B (*Orzya sativa* L. subsp. *indica*) natural mutations. During grain hull development, the color of the mutant hull changed from straw-white to pink, then purple, and finally brownish-yellow. Color change is an excellent tool for analyzing flavonoid metabolic processes because of similar genetic backgrounds. Therefore, a large-scale flavonoid characterization was performed to investigate the accumulation of flavonoids in tissues to establish whether varying metabolite profiles lead to different pigmentation of the grain hull. Meanwhile, bulked segregant analysis based on deep sequencing (BSA-seq) and gene mapping approaches were performed to map the candidate genes. These findings will increase our understanding of the biosynthesis of rice pigmentation and provide valuable information needed for breeding rice in the future.

## Results and Discussion

### Phenotypic Characterization of the *pg* Mutant

**Fig. 1:**
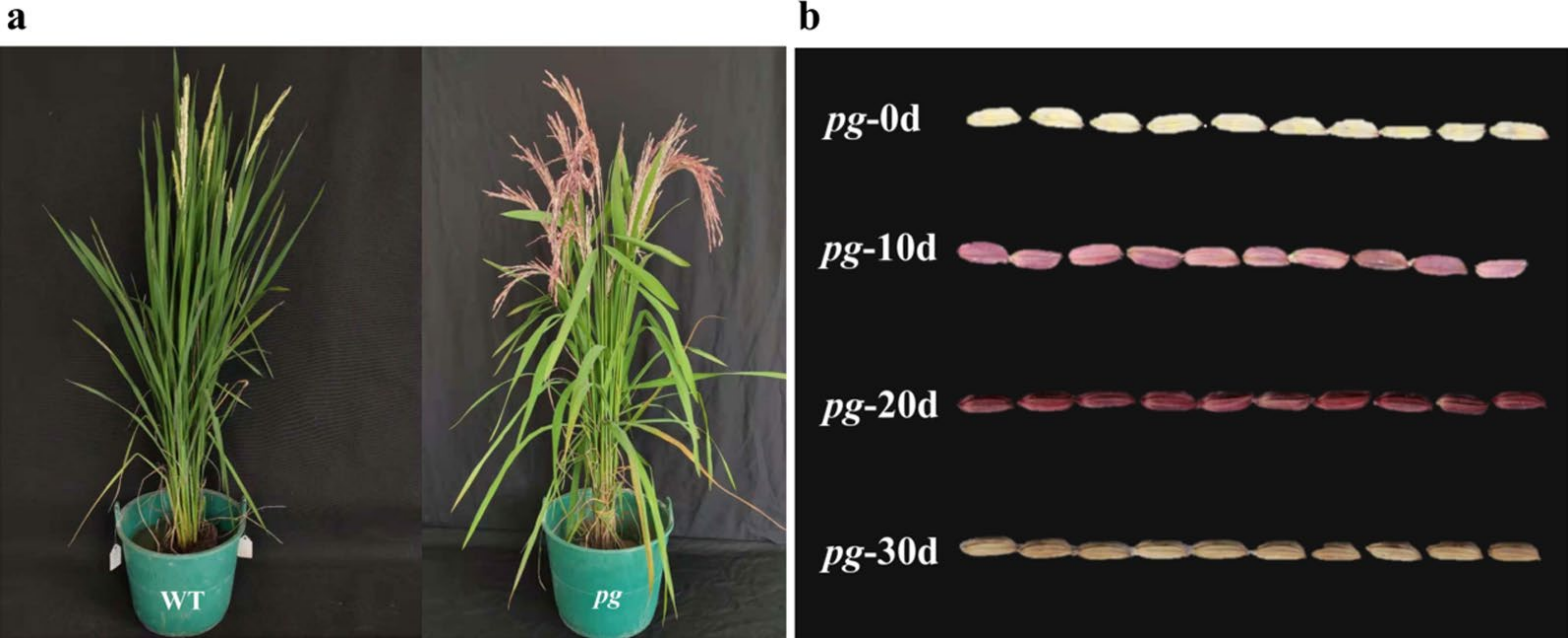
A comparison of the morphology of wild type (IARI 6184B) and the purple gradient grain hull mutant (*pg*). **a** The plants at the heading stage. **b** The hulls of the *pg* mutant at different heading stages

The *pg* mutant plants showed purple gradient grain hulls, whereas the wild-type (WT) hulls were straw-white at the heading stage (Fig. 1a). The hulls of the *pg* mutant were straw-white at the initial heading stage (*pg*-0d), gradually turned pink at 10 days after heading (*pg*-10d), deepened to dark purple at 20 days after heading (*pg*-20d), and finally turned to brownish-yellow at the fully mature stage of the rice grains (30 days) (*pg*-30d) (Fig. 1b). Differences between WT and *pg* mutant were observed in the plant, spikelet, and grain traits, with higher panicle number per plant, higher seed setting rate, lower 1000-grain weight, and lower total grain number per panicle recorded in the *pg* mutant compared with those of WT plants (Table 1). There were no significant differences between the WT and *pg* mutant for single panicle weight, filled grain number per panicle, grain density, average panicle length, and plant height (Table 1).

**Table 1:** Agronomic traits of the wild type IARI 6184B and the purple gradient grain hull mutant (*pg*) plants

Traits	*pg*	Wild type
TGW (g)	19.60 ± 0.91*	22.70 ± 1.48
SPW (g)	1.60 ± 0.32	1.90 ± 0.28
SR (%)	80.43 ± 11.00*	63.71 ± 4.69
FGN (No.)	81.13 ± 13.62	83.87 ± 12.56
TGN (No.)	100.92 ± 10.13*	132.06 ± 21.64
GD (No.)	3.69 ± 0.67	3.95 ± 0.30
PN (No.)	13.00 ± 2.65*	9.00 ± 1.73
PH (cm)	97.23 ± 2.04	98.73 ± 2.41
PL (cm)	22.07 ± 0.82	21.19 ± 1.70

TGW, 1000-grain weight; SPW, Single panicle weight; SR, seed setting rate; FGN, Filled grain number per panicle; TGN, Total grain number per panicle; GD, Grain density; PL, Average panicle length; PN, Panicle number per plant; PH, Plant height

*Represent a significant difference at the 0.05 level by the Student *t*-test. All data represented as mean ± SD

Rice grain hull color is an easily observable trait and is a crucial morphological marker for rice breeding. The main rice hull color mutants are golden yellow (Wang et al. 2017), brown (Shao et al. 2012; Xu et al. 2015), and virescent (Wang et al. 2021), while the black mutant is one of the common wild rice traits (Zhu et al. 2011). The *pg* mutant is a novel rice hull color with ornamental value for the integration and development of agriculture and tourism. The accumulation of anthocyanins and the lack of lignin synthesis both contributed to the change of rice hull color, but the lack of lignin synthesis also caused the variation of internode color (Wang et al. 2017; Zhang et al. 2006). Therefore, the variation in *pg* hull may be due to the accumulation of anthocyanin derivatives.

### Flavonoids Metabolic Profiling of the *pg* Mutant

Flavonoids comprise the majority of pigment molecules in rice hulls. A new metabolomic strategy based on UPLC-MS/MS was used to identify and estimate flavonoid metabolism (Chen et al. 2013; Peng et al. 2017), to assess the changes in flavonoid metabolites of *pg* mutant hulls at different developmental stages. Results revealed 217 flavonoids, including 46 flavonols, 73 flavones, 5 isoflavones, 18 anthocyanins, 40 flavone C-glycosides, 21 dihydroflavonols, 11 flavanols, and 3 chalcones in hulls from four heading stages (Additional file 3: Table S2). Among the 18 anthocyanins, 16 were identified in the straw-white hulls, 18 in the pink and purple hulls, and 17 in the brownish-yellow hulls (Additional file 3: Table S2), suggesting that colorless rice hulls can also synthesize anthocyanins.

**Fig. 2:**
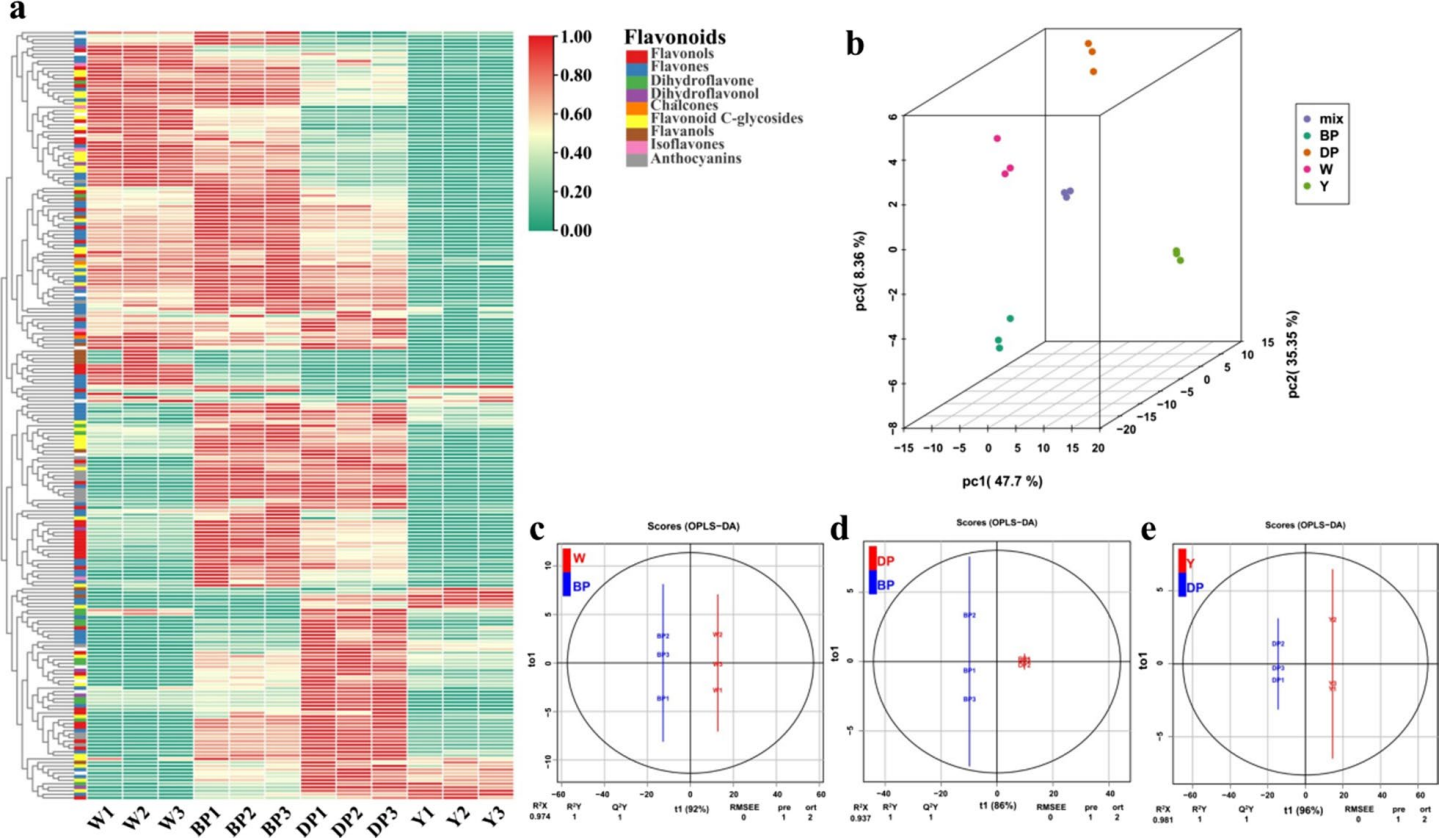
Heat map visualization and principal component analysis (PCA) with orthogonal projection to latent structure discriminant analysis (OPLS-DA) of relative flavonoids in different stages of hull heading. **a** Heat map visualization. **b** In the score plots for PC1 and PC2, strong cohesion was observed within groups, and good separation occurred between heading stages. **c–e** OPLS-DA model plots. W = *pg*-0d; BP = *pg*-10d; DP = *pg*-20d; Y = *pg*-30d

Hierarchical cluster analysis was performed on the above profiles to evaluate differences between metabolic profiles across four developmental stages. The metabolite profile was divided into four major clusters: clusters I, II, III, and IV, representing the accumulation of flavonoids at *pg*-0d, *pg*-10d, *pg*-20d, and *pg*-30d, respectively (Fig. 2a). In addition, principal component analysis (PCA) was conducted to resolve the intrinsic structure of flavonoids variation in the relative content of flavonoids in hulls from four developmental stages. Clear metabolite separation of *pg*-0d, *pg*-10d, *pg*-20d, and *pg*-30d was observed through PCA, indicating significant intergroup specificity of flavonoids metabolites in the hulls of *pg* mutant at different developmental stages (Fig. 2b).

Orthogonal projection to latent structure discriminant analysis (OPLS-DA), a supervised pattern recognition method, enabled visualization and depiction of general variations in metabolism among the four groups. High predictability (Q^2^) and strong goodness of fit (R^2^X, R^2^Y) of OPLS-DA models were observed in the comparison between *pg*-0d and *pg*-10d (R^2^X = 0.974, Q^2^ = 1, R^2^Y = 1), *pg*-20d and *pg*-10d (R^2^X = 0.937, Q^2^ = 1, R^2^Y = 1), and *pg*-30d and *pg*-20d (R^2^X = 0.981, Q^2^ = 1, R^2^Y = 1), suggesting that the model is stable, reliable, and has good discriminant analysis ability (Fig. 2c–e). After 200 permutation test results of the OPLS-DA model, the R^2’^ and Q^2’^ of the new model were smaller than those of the original after Y replacement (Additional file 1: Fig. S1), indicating that the differential metabolites between different groups could be screened according to their variable importance in the project (VIP).

### Identification of Differential Flavonoids Metabolite

To understand the metabolic differences between *pg*-0d, *pg*-10d, *pg*-20d, and *pg*-30d, a differential metabolite screen was run among 217 identified metabolites based on fold change (FC) and VIP (FC ≥ 2 or ≤ 0.5 and VIP ≥ 1.0 were set as thresholds). Based on this criterion, there were 53 differential metabolites (50 upregulated and 3 downregulated) between *pg*-0d and *pg*-10d, 47 (25 upregulated and 22 downregulated) between *pg*-10d and *pg*-20d, 43 (all downregulated) between *pg*-20d and *pg*-30d, and 48 (19 upregulated and 29 downregulated) between *pg*-0d and *pg*-30d (Additional file 1: Fig. S2).

**Fig. 3:**
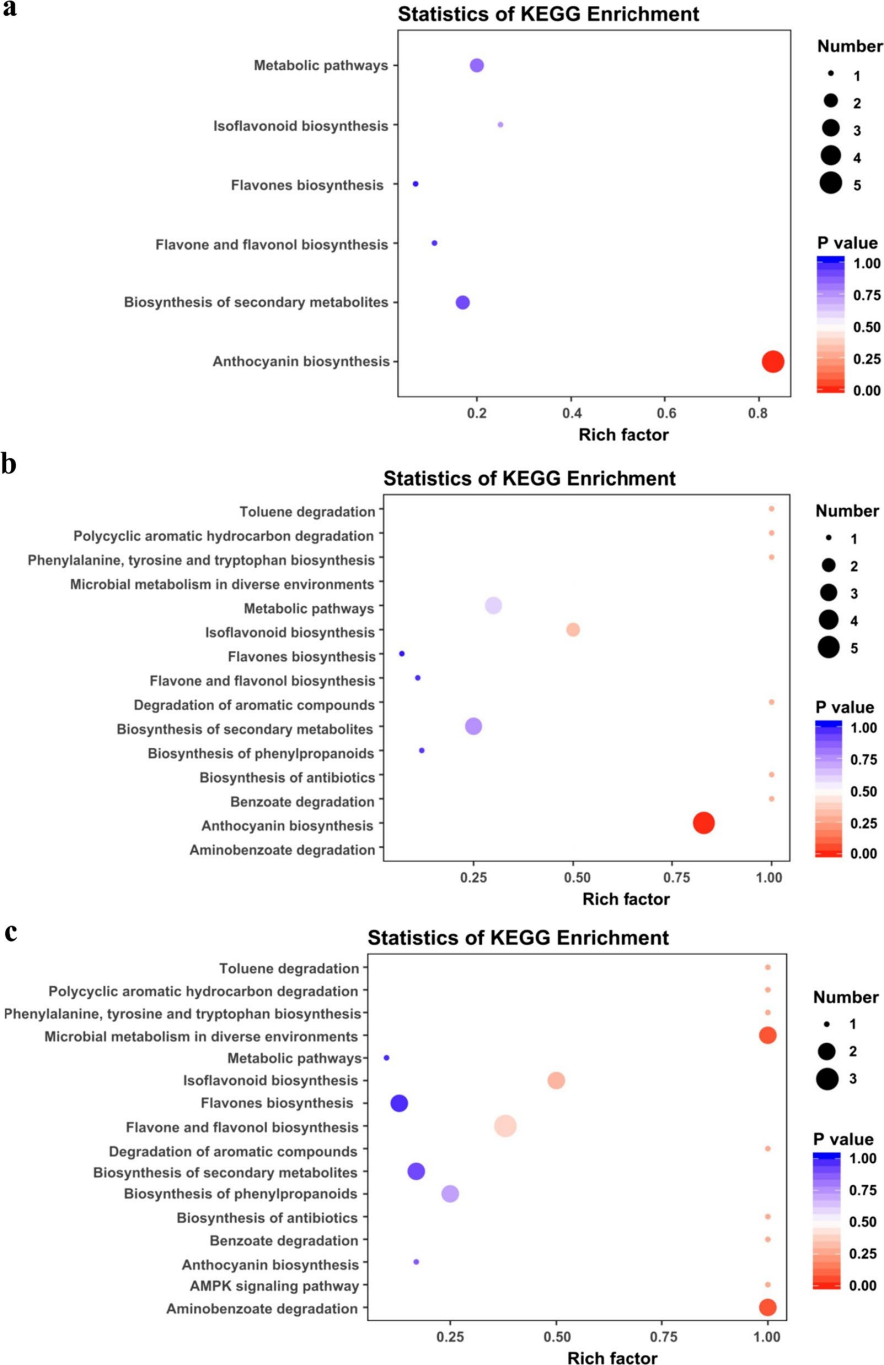
Comparative enrichment of differential metabolites in distinct Kyoto Encyclopedia of Genes and Genome (KEGG) pathways. The metabolic pathways associated with metabolites in *pg*-10d (**a**), *pg*-20d (**b**), and *pg*-30d (**c**) differed from those in *pg*-0d. A hypergeometric distribution was used to compute *p*-values for enrichment

The differential metabolites between the four developmental stages were mapped using the Kyoto Encyclopedia of Genes and Genomes (KEGG, http://www.genome.jp/kegg/). Anthocyanin synthesis was the most significantly enriched metabolic pathway in *pg*-10d and *pg*-20d groups, accounting for 31.25% and 29.41%, respectively (Fig. 3a, b). Furthermore, in the *pg*-10d and *pg*-20d groups, delphin chloride, peonidin 3-O-glucoside, cyanidin 3-O-rutinoside, cyanidin 3-O-glucoside, and pelargonidin 3-O-glucoside metabolites were upregulated in the anthocyanin synthesis pathway compared to the *pg*-0d group. However, in the *pg*-30d group, only one metabolite was associated with anthocyanin synthesis (Fig. 3c), indicating that anthocyanin metabolism was the main cause of the color change in the pink and purple hulls.

### Comprehensive Comparison of the Metabolism of Flavonoids in Grain Hulls

Anthocyanins are a branch of flavonoid biosynthesis. Colored flavonoids (flavanols, isoflavonoids, and flavones) and their glycosides are responsible for coloring leaves, fruits, and flowers (Zhang et al. 2020; Berni et al. 2021; Yang et al. 2022). Analysis of the relative contents of the top 20 metabolites in rice hulls at different developmental stages revealed that tricin 4′-O-β-guaiacylglycerol had the highest relative content in *pg*-0d (straw-white) (flavones, 53.4 × 10^6^) and *pg*-30d (brownish-yellow) (flavones, 28.5 × 10^6^), followed by salcolin A [flavones, 47.7 × 10^6^ (*pg*-0d, straw-white) and 27.6 × 10^6^ (*pg*-30d, brownish-yellow)] (Table 2). However, cyanidin O-syringic acid was the most abundant substance in *pg*-10d (pink) (anthocyanins, 66.2 × 10^6^) and *pg*-20d (purple) (anthocyanins, 68.0 × 10^6^), followed by tricin 4′-O-β-guaiacylglycerol [flavones, 60.7 × 10^6^ (*pg*-10d, pink) and 49.4 × 10^6^ (*pg*-20d, purple)] (Table 2). Previous studies found that cyanidin O-syringic acid was the most abundant anthocyanin in red- and purple-colored vegetables and fruits, such as kiwifruit, kiwiberry, and radishes (Montefiori et al. 2009; Yu et al. 2020; Zhang et al. 2020). However, contrary to previous studies, cyanidin 3-O-glucoside and peonidin 3-O-glucoside were the two major anthocyanins found in the pericarp of black rice (Lee 2010; Shao et al. 2013; Das et al. 2020).

**Table 2:** Relative contents of the top 20 metabolites in rice grain hulls at different heading stages

Class	Compounds	Relative Contents (× 10^6^)			
		*pg*-0d	*pg*-10d	*pg*-20d	*pg*-30d
Flavone C-glycosides	HoMoorientin	19.8 ± 0.21	20.1 ± 0.4	11.5 ± 0.28	5.29 ± 0.21
	Orientin	18.2 ± 1.29	20.1 ± 0.64	13.5 ± 0.61	7.1 ± 0.49
	8-C-Hexosyl-apigenin O-feruloylhexoside	11.4 ± 0.37	18.3 ± 0.53	15.5 ± 1.03	8.64 ± 1.0
	Apigenin-8-C-glucoside	17.7 ± 0.93	14.1 ± 0.77	–	–
	Genistein 8-C-glucoside	27.9 ± 1.08	21.8 ± 2.1	–	–
	Isovitexin	31.6 ± 1.19	24.3 ± 1.5	–	–
	Swertiajaponin	–	–	–	4.06 ± 0.15
Flavonols	Rutin	–	13.8 ± 0.81	16.2 ± 1.15	3.91 ± 0.19
	Isorhamnetin-3-O-rutinoside	–	17.1 ± 0.34	17.7 ± 1.49	5.21 ± 0.39
	Bioquercetin	–	16.9 ± 1.55	20.6 ± 1.86	4.80 ± 0.37
	Quercetin-O-feruloyl-Pentoside	–	30.9 ± 0.68	38.9 ± 1.44	11.5 ± 0.18
Flavones	Tricin O-saccharic acid	–	–	10.2 ± 1.03	5.89 ± 0.47
	Diosmetin-6-C-glucoside	–	–	10.1 ± 1.29	–
	Tricin-O-Hexoside-O-rhamnoside	5.62 ± 0.73	–	12.0 ± 0.90	15.1 ± 0.45
	Tricin 5-O-rutinoside	7.49 ± 0.59	12.9 ± 0.17	12.2 ± 0.59	9.02 ± 0.17
	Salcolin B	14.6 ± 0.62	17.4 ± 1.23	12.9 ± 0.74	6.32 ± 0.26
	Di-C,C-hexosyl-apigenin	17.3 ± 0.5	24.1 ± 1	22.4 ± 0.41	9.17 ± 0.34
	Tricin 4'-O-(β-guaiacylglyceryl) ether 7-O-hexoside	27.6 ± 1.34	34.3 ± 2.03	29.9 ± 1.06	13.9 ± 0.74
	Tricin O-malonylhexoside	38.2 ± 0.79	53.5 ± 1.81	39.2 ± 1.62	13.5 ± 0.39
	Salcolin A	47.7 ± 1.09	51.2 ± 1.77	43.3 ± 1.41	27.6 ± 0.74
	Tricin 4'-O-β-guaiacylglycerol	53.4 ± 0.64	60.7 ± 3.17	49.4 ± 0.87	28.5 ± 0.41
	Tricin	12.4 ± 0.37	12.0 ± 0.95	–	4.83 ± 0.5
	Tricin 4'-O-syringyl alcohol	9.92 ± 0.56	–	–	3.96 ± 0.12
Anthocyanins	Jaceosidin	6.67 ± 0.29	–	–	–
	Cyanidin O-syringic acid	9.40 ± 0.26	66.2 ± 5.57	68.0 ± 2.72	3.85 ± 0.39
	Cyanidin 3-rutinoside	7.11 ± 0.28	52.0 ± 1.40	43.4 ± 2.07	–
	Cyanidin 3-O-glucoside	–	–	8.47 ± 0.49	–
Flavanols	Gallic acid O-feruloyl-O-hexosyl-O-hexoside	7.59 ± 0.75	–	–	–

“–” represents the non-top 20 metabolites

**Fig. 4:**
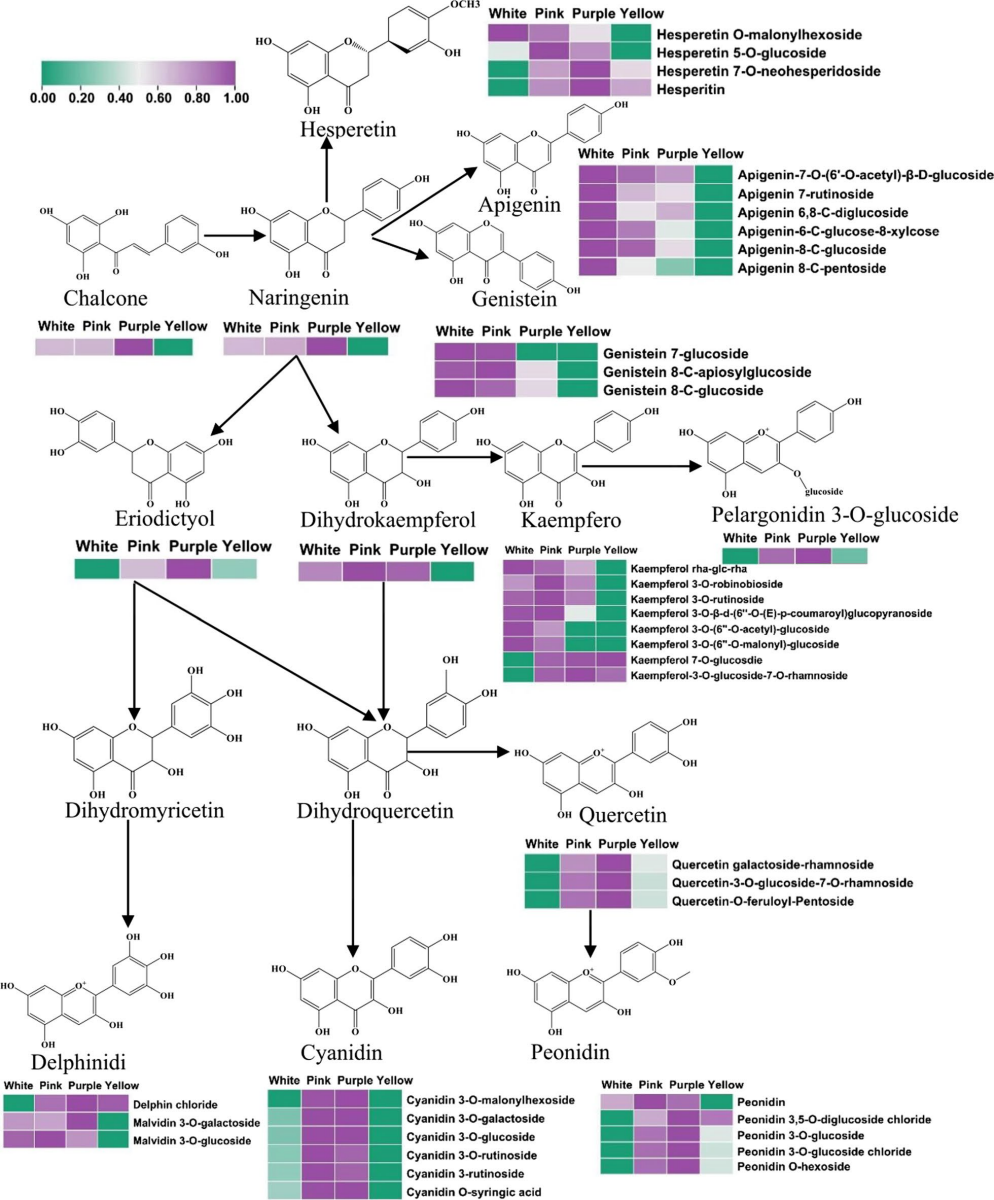
Heat map of flavonoids biosynthesis pathway, constructed by combining Kyoto Encyclopedia of Genes and Genome (KEGG) pathways and literature references. Each colored row represents the log_10_ (content) of a certain metabolite

Chalcone synthase catalyzes the combination of *p*-coumaroyl-CoA with three acetate units from malonyl-CoA to produce tetrahydroxychalcone (anthocyanin skeleton) (Saigo et al. 2020; Xia et al. 2021). However, the conversion of the yellow-colored tetrahydroxychalcone into colorless naringenin is catalyzed by the chalcone isomerase enzyme. In addition, naringenin subsequently produces apigenin, hesperetin, genistein, and dihydrokaempferol via FNS, MTs, and F3H respectively (Cappellini et al. 2021). Finally, the three colorless substances are converted to flavones, flavonols, and anthocyanins (Cappellini et al. 2021). As shown in Fig. 4, tetrahydroxychalcone and naringenin contents were similar in straw-white and pink hulls but were upregulated in purple hulls. The relative contents of hesperetin O-malonylhexoside, apigenin-7-O-(6′-O-acetyl)-β-D-glucoside, apigenin 7-rutinoside, apigenin-6-C-glucose-8-xylcose, apigenin-8-C-glucoside, apigenin 8-C-pentoside, genistein 7-glucoside, genistein 8-C-apiosylglucoside, genistein 8-C-glucoside, and kaempferol 3-O derivatives were consistently downregulated during hull development (Fig. 4). In contrast, kaempferol 7-O-glucoside was consistently upregulated during hull development. Furthermore, 12 anthocyanins (6 cyanidin, 4 peonidin, 1 pelargonidin, and 1 delphin) were upregulated in pink, purple, and brownish-yellow hulls (Fig. 4; Additional file 4: Table S3). Cyanidin 3-O-malonylhexoside and delphin chloride were upregulated more than 1000-fold. However, cyanidin 3-O-malonylhexoside was abundant only in pink and purple hulls, indicating that the change in cyanidin 3-O-malonylhexose led to the *pg* phenotype of rice hulls (Fig. 4; Additional file 4: Table S3). The results indicated that the synthesis pathways of flavones and flavonols were mimicked during the development of *pg* mutant hulls; in contrast, the synthesis pathways related to cyanidin were promoted.

### Genetic and BSA Correlation Analysis

To clarify the *pg* mutant regulatory genes, BSA-seq was used to perform gene mapping. All F_1_ plants derived from the crossing of the *pg* mutant and the Ziyedao (green grain hull) (*Oryza sativa* L. subsp. *japonica*) uniformly displayed *pg* mutant hulls. Among 557 F_2_ plants, 426 were purple gradient, and 131 showed green hulls. As segregation in the F_2_ population displayed a good fit of 3:1 ratio (χ^2^_(3:1)_ = 0.652 < χ^2^_(0.05)_ = 3.84), the *pg* grain hull trait in *pg* mutant was controlled by one nuclear dominant gene.

**Fig. 5:**
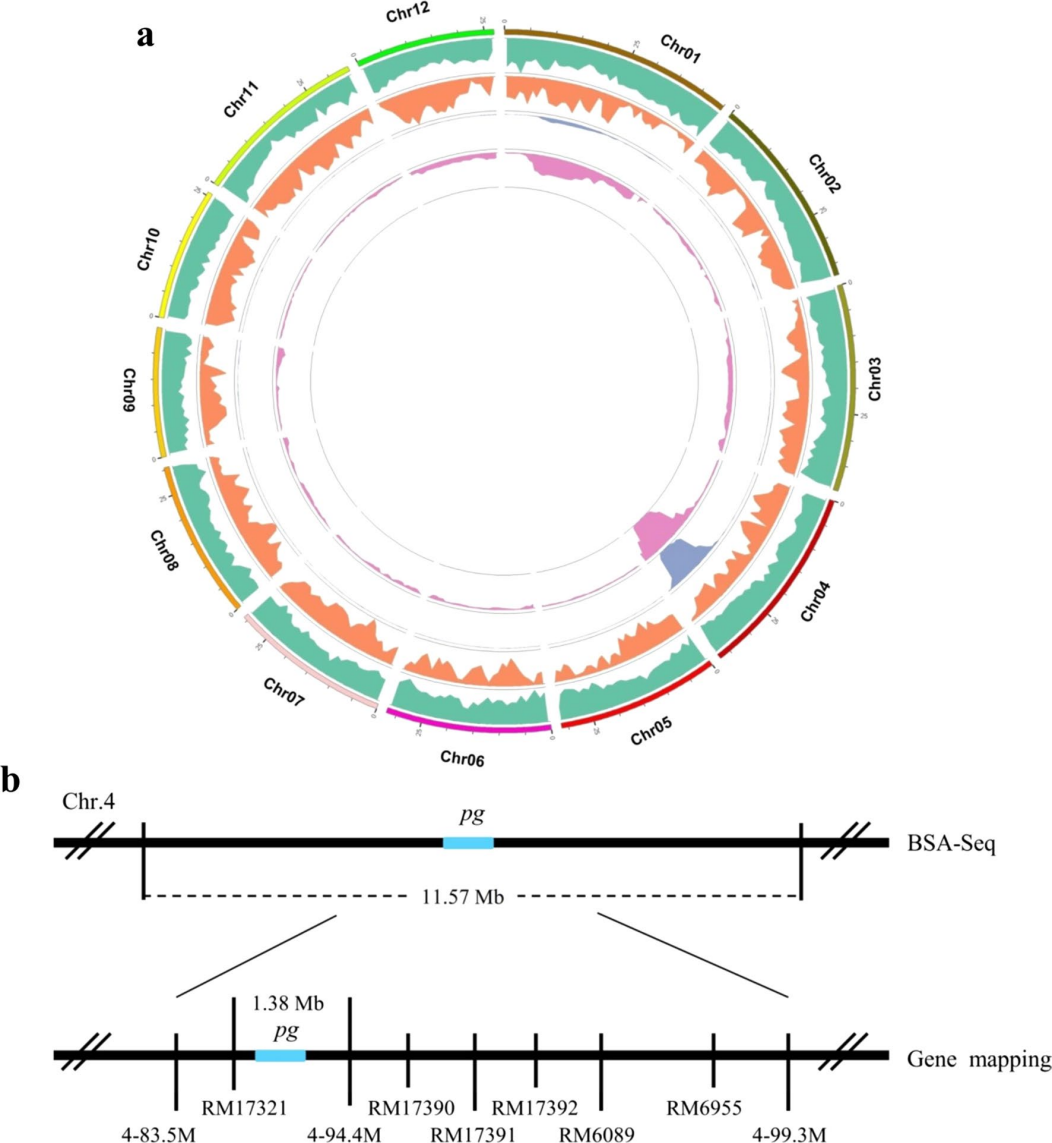
Map position of the *pg* gene. **a** The *pg* gene was mapped to a 11.57 Mb interval on chromosome 4 by the bulked segregant analysis based on deep sequencing (BSA-Seq) approach. **b** The *pg* gene was further mapped to a 1.38 Mb interval by gene mapping approach

Furthermore, 2,072,328 SNPs were obtained by simplified genome sequencing of the *pg* mutant and green hull DNA pools. After eliminating the less reliable markers, 898,837 high-quality SNPs with uniform coverage of 12 rice chromosomes were obtained. The ΔSNP index was then fitted using the DISTANCE method, and the association threshold was obtained by combining the theoretical segregation ratio of the population to 0.667. As a result, one interval was associated with chromosome 4, 14.22 Mb long, containing 2209 genes, of which 789 had non-synonymous mutation loci (Fig. 5a). Furthermore, the ED values were analyzed by counting the depth of each base in the different mixing pools and calculating the ED values for each site. Finally, the median + 3SD = 0.60 of the fitted values for all loci was taken as the association threshold for the analysis. Based on the association threshold, one interval 11.57 Mb in length was obtained on chromosome 4, containing 1,847 genes, of which 747 had non-synonymous mutation loci (Fig. 5a).

### Gene Mapping of the *pg* Mutant

The screening of molecular markers within the BSA association interval for genotypic validation of both parents and the F_2_ population showed that the gene for *pg* hulls was detected on chromosome 4 between 4–83.5 M and 4–99.3 M. For further mapping, the plants with purple gradient hulls were used to trap the target gene by narrowing the distance between 4–83.5 M and 4–99.3 M. Finally, the target gene was narrowed down to a interval between markers RM17321 and 4–94.4 M. The genetic distance between the two markers was about 2.0 cM, and the physical distance was approximately 1.38 Mb (Fig. 5b). The mapped region contained 154 putative genes, of which 4 genes, including *Os04g0557200* encoding an anthocyanin regulatory R-S protein, *Os04g0557500* encoding a bHLH transcription factor, *Os04g0557800* similar to a R-type bHLH protein, and *Os04g0565900* containing a bHLH domain were predicted to be associated with flavonoid synthesis.

The *C*-*S*-*A* gene system regulates rice hull color, involving *C1* encoding the R2R3 MYB transcription factor, *S1* encoding the bHLH protein and functioning tissue-specific, and *A1* encoding a dihydroflavonol reductase has been proposed (Sun et al. 2018; Qiao et al. 2021). A protein–protein interaction occurs between the bHLH and R2R3 MYB domains, activating downstream genes in the structural anthocyanin biosynthesis pathway (Kim et al. 2021; Kong et al. 2012). Alterations to the HLH domain can affect protein–protein interactions between HLH and any other protein, enhancing or reducing the activities of bHLH proteins (Kim et al. 2021). In this study, BSA-seq and gene mapping approaches were used to map the candidate gene to a 1.38 Mb region on chromosome 4. In the mapped region, four genes, *Os04g0557200*, *Os04g0557500*, *Os04g0557800*, and *Os04g0565900*, were associated with flavonoid synthesis. *Os04g0557200*, encoding an anthocyanin regulatory R-S protein, was expressed specifically in fills, buds, and mammary grains (Wang et al. 2015). *Os04g0557500* is presumed to be a candidate gene for hull-specific pigmentation (Sun et al. 2018). *C1* interacts with *S1* and activates *A1* expression resulting in cyanogenic 3-O-glucoside accumulation (Sun et al. 2018). However, in our study, cyanidin O-syringic acid showed the highest pigmentation in *pg* grain hulls. Therefore, further studies are needed to validate the regulatory genes of the *pg* grain hulls.

## Conclusion

A novel mutant of rice purple gradient grain hull color was reported in this study. We analyzed the phenotypic and flavonoid metabolic profile differences among different hull development stages. We have shown that the accumulation of anthocyanin derivatives was the main reason for the formation of purple and pink hulls. In addition, we explored the composition and content of the upstream flavonoid metabolites of anthocyanins, and the results indicated that tetrahydroxychalcone and naringenin were mainly used for the synthesis of cyanidin derivatives, including cyanidin 3-O-glucoside, cyanidin O-syringic acid, and cyanidin 3-O-malonylhexoside. Combined with flavonoid metabolism, the mapping strategy screened out four candidate genes, *Os04g0557200*, *Os04g0557500*, *Os04g0557800*, and *Os04g0565900*, which may be responsible for anthocyanin accumulation in *pg* hull mutant.

## Methods

### Plant Materials and Measurement of Phenotypic Traits

The rice purple gradient grain hull mutant (*pg*) was naturally mutated from a straw-white grain hull rice variety IARI 6184B (PI 353693) (*Oryza sativa* L. subsp. *indica*), introduced to China from India. A stable *pg* mutant was crossed with a green grain hull variety Ziyedao (*Oryza sativa* L. subsp. *japonica*) to generate first-generation (F_1_) plants for phenotypic segregation analysis and genetic mapping. The F_1_ plants were then selfed to produce a second-generation (F_2_) population. All parents and F_2_ plants were grown in paddy fields at the Rice Research Institute, Jiangxi Academy of Agricultural Sciences, Jiangxi, China. Agronomic traits, such as single panicle weight, average panicle length, plant height, grain density, 1000-grain weight, filled grain number per panicle, panicle number per plant, total grain number per panicle, and seed setting rate were measured.

### Extraction of Flavonoids

Rice mutant hulls at 0 (*pg*-0d), 10 (*pg*-10d), 20 (*pg*-20d), and 30 days (*pg*-30d) after heading were obtained for flavonoids analysis. A grinder (MM 400; Retsch, Munich, Germany) was used to grind the freeze-dried rice hulls to powder for 1.5 min at 30 Hz. The powder (100 mg) was dissolved in 1.0 ml of 70% aqueous methanol and extracted at 4 °C for 12 h. The supernatant was centrifuged at 10,000 rpm for 10 min, filtered through a microporous membrane (SCAA-104, 0.22 µm pore size; ANPEL laboratory technologies, Shanghai, China), and transferred to a sample bottle for analysis using ultra-performance liquid chromatography-tandem mass spectrometry (UPLC-MS/MS).

### UPLC-MS/MS Conditions

The UPLC-MS/MS (CBM30A, Shimadzu Corporation, Kyoto, Japan) and electrospray ionization tandem mass spectrometry systems (4500 QTRAP, Applied Biosystems, Waltham, MA, USA) were used to analyze the sample extracts. Each sample (*pg*-0d, *pg*-10d, *pg*-20d, and *pg*-30d) was replicated thrice. First, 5 µL of each sample was injected into an Acquity UPLC high strength silica T3 C18 column (2.1 × 100 mm, with a pore size of 1.8 µm) (Acquity; Waters, Milford, MA, USA), and the column was kept at 40 °C. Next, the mobile phase was maintained at 0.4 mL/min throughout the gradient. Eluent A was water containing 0.04% acetic acid, and eluent B was acetonitrile containing 0.04% acetic acid. The gradient programs were applied as follows: 100:0 V(A)/V(B) at 0 min, 5:95 V(A)/V(B) at 11.0 min, 5:95 V(A)/V(B) at 12.0 min, 95:5 V(A)/V(B) at 12.1 min, and 95:5 V(A)/V(B) at 15.0 min. Quality control samples were injected five times to increase accuracy. The data were collected using a triple quadrupole tandem mass spectrometer with multiple reaction monitoring (Oxford Instruments, Abingdon, UK) and processed using Analyst 1.6.1 software (Sciex, Framingham, MA, USA). The mass spectrometry conditions were set following the method described by Chen et al. (2013).

### Construction of Purple and Green Hull Extreme Pools

Plants from the F_2_ population formed by crossing *pg* mutant (purple gradient grain hull) with Ziyedao (green grain hull) were visually counted for genetic analysis. The segregation ratio in purple- and green-hulled plants was analyzed. The DNA of the fresh leaves was extracted using the cetyltrimethylammonium bromide (CTAB) method. Based on the phenotypic identification of the F_2_ population, genomic DNA pools of the two parental and two F_2_ pools with extreme phenotypes were constructed for the BSA-Seq analysis, including the *pg* mutant, Ziyedao, purple gradient grain hull (24 F_2_ individuals), and green hull (24 F_2_ individuals) pools. DNA sequencing was performed using an Illumina HiSeq™ 2500 platform (Illumina, San Diego, CA, USA). The sequencing depth was approximately 30 times more than that of the rice genome (Beijing Biomarker Biotechnology Co., Beijing, China).

After the raw sequencing data were stripped of junctions and low-quality sequences, they were aligned with the reference genome (*Oryza sativa*: MH63RS3), and the results were used to remove duplicate sequences. Based on the localization results of clean reads, pre-processing such as mark duplicates, local realignment, base recalibration, and single nucleotide polymorphisms (SNPs) were performed using Picard command-line tools and genome analysis toolkit (GATK) (McKenna et al. 2010).

### Mapping of the *pg* Gene

The Euclidean distance (ED) algorithm was used to identify significant differences between markers of purple and green hulls. After eliminating background noise with the third power of ED, SNPs number methods (Takagi et al. 2013) were used to fit the ED values to the correlation value and select the interval above the threshold value as the interval associated with hull color genes. Fine mapping was performed after receiving directions for the candidate regions to narrow down candidates by designing additional simple sequence repeats (SSR) and insertion/deletion (InDel) primers for the candidate regions. The primers used for gene mapping were listed in Additional file 2: Table S1. The Rice Genome Annotation Project database (http://rice.uga.edu/) and NCBI database (https://www.ncbi.nlm.nih.gov/) were searched for functional annotations of genes within the candidate region.

### Data Processing and Multivariate Statistical Analysis

The Fisher’s least significant difference test (*p* < 0.05, *p* < 0.01) was used to determine significant differences. The mean standard deviation (SD) was calculated based on at least three biological replicates/treatments. Metabolites data were integrated and corrected using Analyst 1.6.3 software and multiple reaction monitoring. Significantly regulated metabolites were determined at *p* < 0.05 and absolute log_2_FC (fold change) ≥ 1. A heatmap was drawn using TBtools.

## Supplementary Information

**Additional file 1.** **Fig. S1.** Orthogonal projection to latent structure discriminant analysis (OPLS-DA) model verification diagram between BP, DP and Y. The horizontal lines represent R2 and Q^2^ of the original model, and the red and blue dots represent R^2’^ and Q^2’^ of the model after Y substitution, respectively. BP = *pg*-10d; DP = *pg*-20d; Y = *pg*-30d. **Fig. S2.** Heat map visualization of relative flavonoids in the different stages of hulls filling. W = *pg*-0d; BP = *pg*-10d; DP = *pg*-20d; Y = *pg*-30d.

**Additional file 2.** **Table S1.** Molecular markers used for gene mapping in this study.

**Additional file 3.** **Table S2.** The relative content of the identified flavonoids in four filling stages.

**Additional file 4.** **Table S3.** The relative contents of up-regulation metabolites and FC in rice hulls at different stages.

## Data Availability

Datasets generated in the current study are available from the corresponding author upon reasonable request.
