# Supplementary material for: Flavonoid Metabolic Profiles and Gene Mapping of Rice (Oryza sativa L.) Purple Gradient Grain Hulls
Source: Rice (N Y). 2022 Aug 8;15:43. doi: 10.1186/s12284-022-00589-x (PMC9357590; doi:10.1186/s12284-022-00589-x)
Supplement: Supplementary file 1 — Additional file 1. Fig. S1. Orthogonal projection to latent structure discriminant analysis (OPLS-DA) model verification diagram between BP, DP and Y. The horizontal lines represent R2 and Q2 of the original model, and the red and blue dots represent R2’ and Q2’ of the model after Y substitution, respectively. BP = pg-10d; DP = pg-20d; Y = pg-30d. Fig. S2. Heat map visualization of relative flavonoids in the different stages of hulls filling. W = pg-0d; BP = pg-10d; DP = pg-20d; Y = pg-30d. [file 12284_2022_589_MOESM1_ESM.docx]

Supporting Information

**Flavonoid Metabolic Profiles and Gene Mapping of Rice (Oryza sativa L.) Purple Gradient Grain Hulls**

Fantao Zhang^1^, Limin Yang^1^, Wenxue Huang^1^, Xiangdong Luo^1^, Jiankun Xie^1^, Biaolin Hu^2*^ and Yaling Chen^1*^

^1^Laboratory of Plant Genetic Improvement and Biotechnology, College of Life Sciences, Jiangxi Normal University, No 99, Ziyang Road, Nanchang 330022, Jiangxi, China

^2^Rice Research Institute, Jiangxi Academy of Agricultural Sciences/

National Engineering Laboratory for Rice (Nanchang), No 1738, Liangtangbei Road, Nanchang 330200, Jiangxi, China

*Correspondence: hubiaolin992@126.com; [yaqing620@163.com](mailto:yaqing620@163.com)


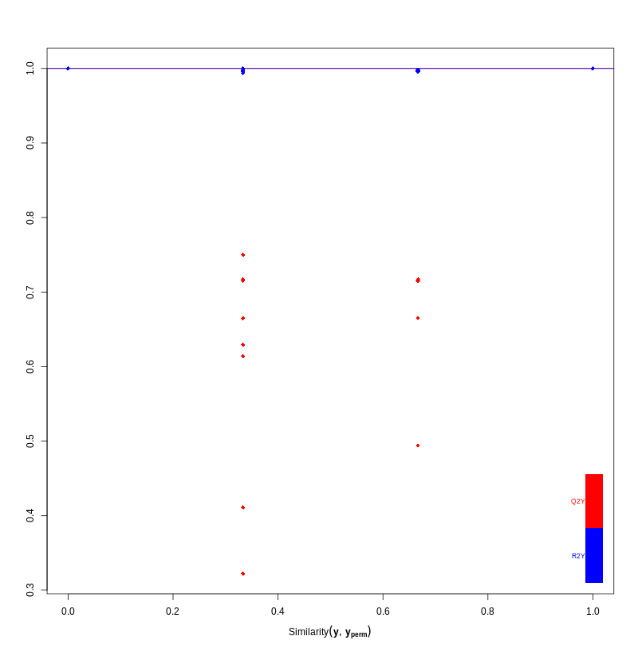

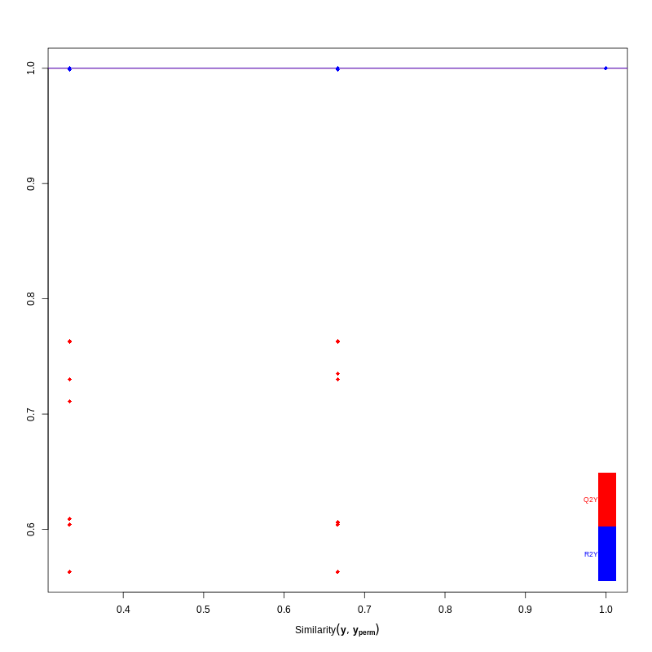


**Fig. S1** Orthogonal projection to latent structure discriminant analysis (OPLS-DA) model verification diagram between BP, DP and Y. The horizontal lines represent R^2^ and Q^2^ of the original model, and the red and blue dots represent R^2’^ and Q^2’^ of the model after Y substitution, respectively. BP = *pg*-10d; DP = *pg*-20d; Y = *pg*-30d

**b**

**a**


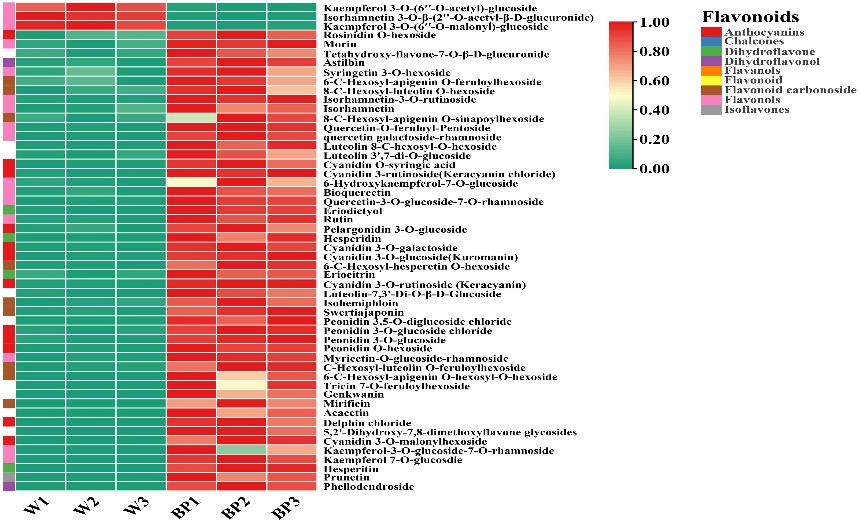

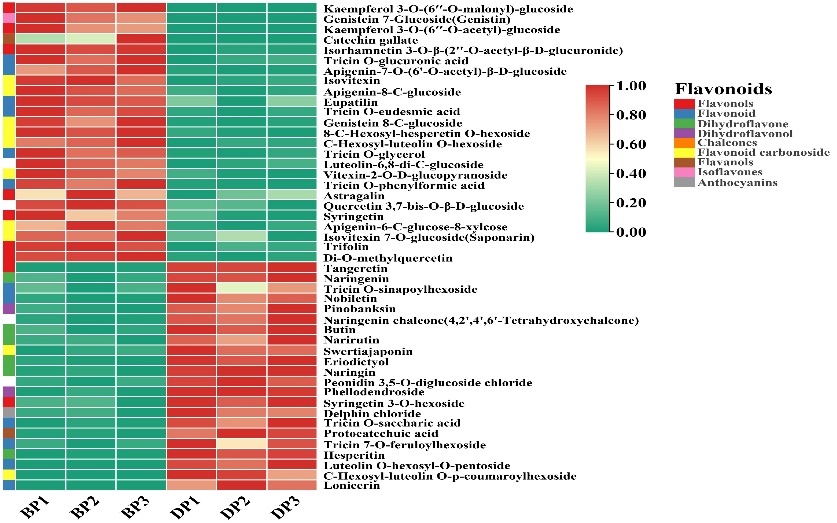


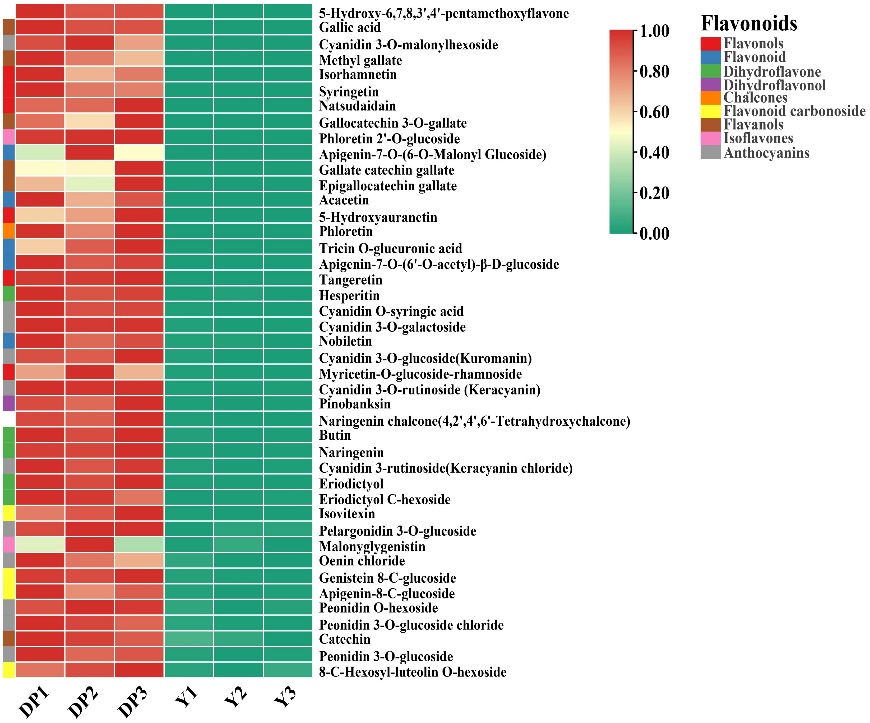

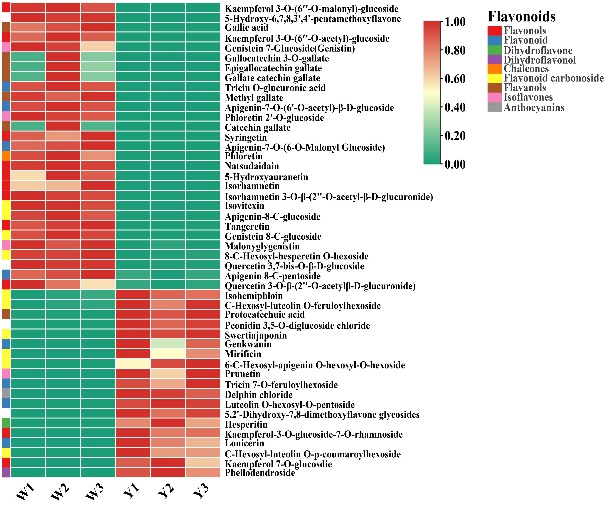


**c**

**d**

**Fig. S2** Heat map visualization of relative flavonoids in the different stages of hulls filling. W= *pg*-0d; BP=*pg*-10d; DP=*pg*-20d; Y=*pg*-30d
