# Supplementary material for: Flavonoid Metabolic Profiles and Gene Mapping of Rice (Oryza sativa L.) Purple Gradient Grain Hulls
Source: Rice (N Y). 2022 Aug 8;15:43. doi: 10.1186/s12284-022-00589-x (PMC9357590; doi:10.1186/s12284-022-00589-x)
Supplement: Supplementary file 2 — Additional file 2. Table S1. Molecular markers used for gene mapping in this study. [file 12284_2022_589_MOESM2_ESM.docx]

Supporting Information

**Flavonoid Metabolic Profiles and Gene Mapping of Rice (Oryza sativa L.) Purple Gradient Grain Hulls**

Fantao Zhang^1^, Limin Yang^1^, Wenxue Huang^1^, Xiangdong Luo^1^, Jiankun Xie^1^, Biaolin Hu^2*^ and Yaling Chen^1*^

^1^Laboratory of Plant Genetic Improvement and Biotechnology, College of Life Sciences, Jiangxi Normal University, No 99, Ziyang Road, Nanchang 330022, Jiangxi, China

^2^Rice Research Institute, Jiangxi Academy of Agricultural Sciences/

National Engineering Laboratory for Rice (Nanchang), No 1738, Liangtangbei Road, Nanchang 330200, Jiangxi, China

*Correspondence: hubiaolin992@126.com; [yaqing620@163.com](mailto:yaqing620@163.com)

**Table S1** Molecular markers used for gene mapping in this study

| **Name** | **F Primer** | **R Primer** | **Location** |
| --- | --- | --- | --- |
| 4-83.5M | cggtgttggcggcttggtgtagt | gatgtttcccttcgtggtttctt | 27,165,630 |
| RM17321 | aaagacaaccgtgaggctagtgg | cgatgatgtacatgggcactcc | 27,697,771 |
| 4-94.4M | gcaatgtcagttcctgatttgt | aaaggacgagcacaacataccc | 29,076,294 |
| RM17390 | ggcatttcttggttagaggatgc | tttctagaccatgagagcgaacg | 29,433,147 |
| RM17391 | actttgctctgaacttgcagtgg | gctagctgctatcaggattcacg | 29,438,311 |
| RM17392 | tcgagcttgtcggtgtcagc | ctcatgggcaagtgattgtttcc | 29,486,470 |
| RM6089 | cgatggccagcgtgatctcc | ccaccgaatcgaataaccacaagc | 29,563,534 |
| RM6955 | aagatccaccgtcatcctctgc | ggtgtgatgctacttgtcagaaacg | 29,781,161 |
| 4-99.3M | gctcctattacaacctactcctg | tagatgtatgggtccgctttagt | 29,962,862 |
