# Supplementary material for: Flavonoid Metabolic Profiles and Gene Mapping of Rice (Oryza sativa L.) Purple Gradient Grain Hulls
Source: Rice (N Y). 2022 Aug 8;15:43. doi: 10.1186/s12284-022-00589-x (PMC9357590; doi:10.1186/s12284-022-00589-x)
Supplement: Supplementary file 4 — Additional file 4. Table S3. The relative contents of up-regulation metabolites and FC in rice hulls at different stages. [file 12284_2022_589_MOESM4_ESM.docx]

Supporting Information

**Flavonoid Metabolic Profiles and Gene Mapping of Rice (Oryza sativa L.) Purple Gradient Grain Hulls**

Fantao Zhang^1^, Limin Yang^1^, Wenxue Huang^1^, Xiangdong Luo^1^, Jiankun Xie^1^, Biaolin Hu^2*^ and Yaling Chen^1*^

^1^Laboratory of Plant Genetic Improvement and Biotechnology, College of Life Sciences, Jiangxi Normal University, No 99, Ziyang Road, Nanchang 330022, Jiangxi, China

^2^Rice Research Institute, Jiangxi Academy of Agricultural Sciences/

National Engineering Laboratory for Rice (Nanchang), No 1738, Liangtangbei Road, Nanchang 330200, Jiangxi, China

*Correspondence: hubiaolin992@126.com; [yaqing620@163.com](mailto:yaqing620@163.com)

**Table S3** The relative contents of up-regulation metabolites and FC in rice hulls at different stages

| **Class** | **Compounds** | **Relative Contents (×10^4^)** | | | | **Fold Change** | | | |
| --- | --- | --- | --- | --- | --- | --- | --- | --- | --- |
|  |  | ***pg*-0d** | ***pg*-10d** | ***pg*-20d** | ***pg*-30d** | ***pg*-10d / *pg*-0d** | ***pg*-20d / *pg*-0d** | ***pg*-30d / *pg*-0d** |  |
| Dihydroflavone | Hesperitin | - | 9.26±0.52 | 94.5±4.27 | 4.02±0.71 | 10300 | 105000 | 4460 |  |
| Dihydroflavonol | Phellodendroside | - | 13.9±0.95 | 42.5±0.41 | 34.5±4.63 | 15500 | 47200 | 38400 |  |
| Anthocyanins | Cyanidin 3-O-malonylhexoside | - | 5.63±0.69 | 7.31±1.17 | - | 6260 | 8120 | - |  |
|  | Delphin chloride | - | 1.37±0.16 | 4.51±0.43 | 2.69±0.17 | 1530 | 5020 | 2980 |  |
|  | Pelargonidin 3-O-glucoside | 3.99±1.03 | 33.1±4.15 | 40.9±1.34 | 6.27±0.70 | 8.31 | 10.3 | 1.57 |  |
|  | Cyanidin 3-O-galactoside | 63.1±3.29 | 546±23.8 | 554±7.76 | 32.8±4.39 | 8.65 | 8.78 | 0.519 |  |
|  | Cyanidin 3-rutinoside | 711±28.1 | 5200±140 | 4340±207 | 332±29.8 | 7.31 | 6.09 | 0.466 |  |
|  | Cyanidin O-syringic acid | 940±26.1 | 6620±557 | 6800±272 | 385±39.2 | 7.05 | 7.23 | 0.409 |  |
|  | Cyanidin 3-O-rutinoside | 45.7±2.55 | 422±6.94 | 339±2.37 | 21.2±0.93 | 9.22 | 7.41 | 0.46 |  |
|  | Cyanidin 3-O-glucoside | 98.1±6.21 | 853±28.2 | 847±48.8 | 51.4±7.32 | 8.70 | 8.63 | 0.52 |  |
|  | Peonidin 3-O-glucoside chloride | 17.9±3.75 | 267±11.2 | 360±21.3 | 62.6±6.42 | 14.9 | 20.1 | 3.50 |  |
|  | Peonidin O-hexoside | 16.3±2.83 | 267±14.1 | 367±13.5 | 62.6±7.21 | 16.4 | 22.5 | 3.84 |  |
|  | Peonidin 3-O-glucoside | 17.4±4.27 | 270±10.5 | 387±24.5 | 72.3±3.98 | 15.5 | 22.3 | 4.15 |  |
|  | Peonidin 3,5-O-diglucoside chloride | 1.8±0.11 | 26.8±2.16 | 79.9±3.18 | 48.1±3.30 | 14.9 | 44.3 | 26.7 |  |
| Flavones | Tricin 7-O-feruloylhexoside | - | 0.84±0.27 | 6.27±1.66 | 2.01±0.35 | 928 | 6970 | 2240 |  |
|  | Acacetin | - | 1.30±0.23 | 0.72±0.13 | - | 1440 | 802 | - |  |
|  | Nobiletin | 2.91±0.09 | 4.33±0.13 | 9.71±0.69 | 0.59±0.09 | 1.48 | 3.33 | 0.20 |  |
| Flavonols | Myricetin-O-glucoside-rhamnoside | 16.0±2.59 | 299±11.4 | 280±57.7 | 17.3±2.21 | 18.7 | 17.5 | 1.08 |  |
|  | Tangeretin | 15.6±0.71 | 30.6±0.25 | 62.5±0.97 | 0.62±0.06 | 1.96 | 4.00 | 0.04 |  |
|  | Myricetin-O-glucoside-rhamnoside | 16.0±2.59 | 299±11.4 | 280±57.7 | 17.3±2.21 | 18.7 | 17.5 | 1.08 |  |
| Flavone C-glycosides | 8-C-Hexosyl-luteolin O-hexoside | 0.62±0.11 | 2.60±0.49 | 2.69±0.21 | 0.51±0.08 | 4.23 | 4.37 | 0.83 |  |
